# Detection of early stage pancreatic cancer using 5-hydroxymethylcytosine signatures in circulating cell free DNA

**DOI:** 10.1038/s41467-020-18965-w

**Published:** 2020-10-19

**Authors:** Gulfem D. Guler, Yuhong Ning, Chin-Jen Ku, Tierney Phillips, Erin McCarthy, Christopher K. Ellison, Anna Bergamaschi, Francois Collin, Paul Lloyd, Aaron Scott, Michael Antoine, Wendy Wang, Kim Chau, Alan Ashworth, Stephen R. Quake, Samuel Levy

**Affiliations:** 1 Bluestar Genomics, 185 Berry Street, Lobby 4, Suite 210, San Francisco, CA 94107 USA; 2 Bluestar Genomics, 10578 Science Center Drive Suite 210, San Diego, CA 92121 USA; 3 UCSF Helen Diller Family Comprehensive Cancer Center, San Francisco, CA 94158 USA; 4 Departments of Bioengineering and Applied Physics, Stanford University, Stanford, CA 94304 USA; 5 Chan Zuckerberg Biohub, San Francisco, CA 94158 USA

**Keywords:** Cancer, Epigenomics, Cancer

## Abstract

Pancreatic cancer is often detected late, when curative therapies are no longer possible. Here, we present non-invasive detection of pancreatic ductal adenocarcinoma (PDAC) by 5-hydroxymethylcytosine (5hmC) changes in circulating cell free DNA from a PDAC cohort (*n* = 64) in comparison with a non-cancer cohort (*n* = 243). Differential hydroxymethylation is found in thousands of genes, most significantly in genes related to pancreas development or function (*GATA4*, *GATA6*, *PROX1*, *ONECUT1*, *MEIS2*), and cancer pathogenesis (*YAP1*, *TEAD1*, *PROX1*, *IGF1*). cfDNA hydroxymethylome in PDAC cohort is differentially enriched for genes that are commonly de-regulated in PDAC tumors upon activation of *KRAS* and inactivation of *TP53*. Regularized regression models built using 5hmC densities in genes perform with AUC of 0.92 (discovery dataset, *n* = 79) and 0.92–0.94 (two independent test sets, *n* = 228). Furthermore, tissue-derived 5hmC features can be used to classify PDAC cfDNA (AUC = 0.88). These findings suggest that 5hmC changes enable classification of PDAC even during early stage disease.

## Abstract (web summary)

Circulating DNA detected in plasma can be used for diagnostic purposes. Here, the authors show that the 5-hydroxymethyl cytosine biomarker from plasma-derived cell free DNA can be used to detect early stage pancreatic cancer.

## Introduction

Pancreatic cancer often presents late and has few symptoms, at which point only 10–20% of patients are eligible for surgical resection^1^. Pancreatic ductal adenocarcinoma (PDAC) constitute more than 90% of all pancreatic cancer cases^2^ with the next most common sub-type being neuroendocrine tumors^1^. Tobacco smoking confers a two- to three-fold higher risk of pancreatic cancer, while contributing to approximately 15–30% of cases^1^, with smokers diagnosed 8 to 15 years younger than non-smokers^3,4^. Family history is contributory in ~10% of cases, and germline mutations in genes such as *BRCA2*, *BRCA1*, *CDKN2A*, *ATM*, *STK11*, *PRSS1*, *MLH1* and *PALB2* are associated with pancreatic cancer with variable penetrance^1^. Translational research using genomic and proteomic technologies has provided molecular insights into the pathogenesis and biology of pancreatic cancer but has yet to yield robust diagnostic biomarkers to impact early diagnosis of disease, as reflected by a low overall 5-year survival rate of 10%^1,2^.

The management of PDAC presents physicians with challenges along the entire clinical spectrum, including early detection in high risk individuals, early diagnosis of patients with symptoms or imaging findings, prognostication of outcomes and prediction of therapeutic responsiveness. Collectively, these factors have engendered intensive efforts in translational research to identify and validate biomarkers with sufficient clinical performance metrics to improve decision algorithms and resultant clinical outcomes. Current guidelines in PDAC management are limited to two biomarker recommendations for detecting disease presence assayed in an invasive fashion from cystic fluid. First, carbohydrate antigen 19-9 (CA 19-9) guides surgery decisions, use of adjuvant therapy, or the detection of post-operative tumor recurrence, however, its utility is limited because 10% of patients do not secrete the antigen^5^. Second, carcinoembryonic antigen (CEA) concentration determination from cyst fluid is used to distinguish higher risk mucinous from non-mucinous cysts^6,7^. CA19-9 levels can also be measured by a blood test, however blood levels of CA19-9 were not found to be sensitive or specific enough for reliable detection of pancreatic cancer^8^.

Among the inherited risk factors for pancreatic cancer are genomic mutations such as *BRCA2*, which confers a 3.5-fold higher risk in carriers, with the probability of a germline mutation between 6 and 12% in PDAC patients with a first-degree relative diagnosed with PDAC^9^. Molecular analyses of pancreatic cancer genomes have further revealed activating mutations in *KRAS* and inactivation of *CDKN2A*, *TP53* and *SMAD4*, either through point mutation or copy number changes at >50% population frequency^10–12^. However, mutational heterogeneity, coupled with lack of disease specificity due to pleiotropy render this subset of genes incomplete for the diagnosis of patients. Molecular subtyping of pancreatic tumors using mutational-based data^11^ or gene expression signatures^13–15^ have not yet seen clinical applicability. Other forms of molecular profiling have focused on epigenetics, namely chromatin-based post-translational modifications and the methylation status of cytosine bases in DNA.

Control of DNA state and chromatin regulation have been observed to underpin the onset and progression of oncologic disease^16,17^. DNA methylation status of cytosine bases has been shown to associate with transcriptional regulation of gene expression. DNA methylation in promoters is associated with gene silencing whereas demethylation is associated with gene activation^18^. Gene body methylation, on the other hand, is correlated with increased expression^19^. More recently, detailed understanding of demethylation has been enabled with precision around intermediate states during active demethylation^20,21^. Specifically, discovery of TET enzyme-mediated methyl-cytosine oxidation to 5-hydroxymethyl cytosine (5hmC) has yielded signatures that enable definition of cellular states^22^, as well as identification of cancer in cell free DNA^23–25^.

Molecular signatures in circulating cell free DNA (cfDNA) based on cytosine 5-hydroxymethylation have been shown previously to potentially define the tissue of tumor origin in a variety of disease types^23^. Therefore, we embarked on a case–control study aimed at investigating whether DNA 5hmC signatures were present in the blood of PDAC patients compared to a cohort of non-cancer individuals. We also investigated whether these signatures enable discrimination between cancer and non-cancer patients.

We find that in our study population, PDAC patients possess thousands of genes with an altered hydroxymethyl profile compared to non-cancer individuals. Furthermore, filtering to those genes with the most differentially hydroxymethylated states reveals genes that have been previously implicated in pancreas development or pancreatic cancer. This biologically significant gene set performs well in the construction of predictive models to discriminate PDAC from non-disease, suggesting that the measurement of 5hmC in cfDNA merits further investigation for the detection and classification of PDAC.

## Results

### Clinical cohort and study design

Plasma specimens from 307 subjects with or without a diagnosis of PDAC were collected at multiple institutions in different geographic regions of the United States. These PDAC (*n* = 64) and non-cancer (*n* = 243) patient samples satisfied the study inclusion criteria, which included male and female subject age of minimum 40 years old with a tolerance of 5% of patients younger than 40 years old, as well as confirmed pathologic diagnosis of adenocarcinoma of any subtype at the time of biopsy or surgical resection for subjects in the cancer cohort. The non-cancer cohort was identified as satisfying the study inclusion criteria and patients were specifically negative for any form of cancer. Neither cohort were being treated with medication for disease at the time of blood collection, which was prior to any biopsy or surgical resection in the cancer cohort. Discovery dataset consisted of 41 PDAC and 38 non-cancer samples from and the remaining samples were used for validation. There were no statistically significant differences in subject age, gender or tobacco exposure between the two cohorts used for discovery (Table 1). Early stage PDAC patients (Stages I & II) made up 56% of the PDAC cohort.

**Table 1:** Clinical characteristics of non-cancer and cancer subject cohorts.

	Non-cancer	Cancer
Age^a^		
	64.9	65.5
Gender (%)		
Male	47.4	43.9
Smoking history Status (%)		
Current	13.2	12.2
Former	42.1	39.0
None	44.7	48.8
Stage (%)		
I	NA	24.4
II	NA	31.7
III	NA	14.6
IV	NA	29.3

Other values are percentages of each category in “Non-Cancer” and “Cancer” groups.

^a^Mean of non-cancer and cancer groups.

### Genomic distributions of 5hmC in PDAC and non-cancer cohort

**Fig. 1:**
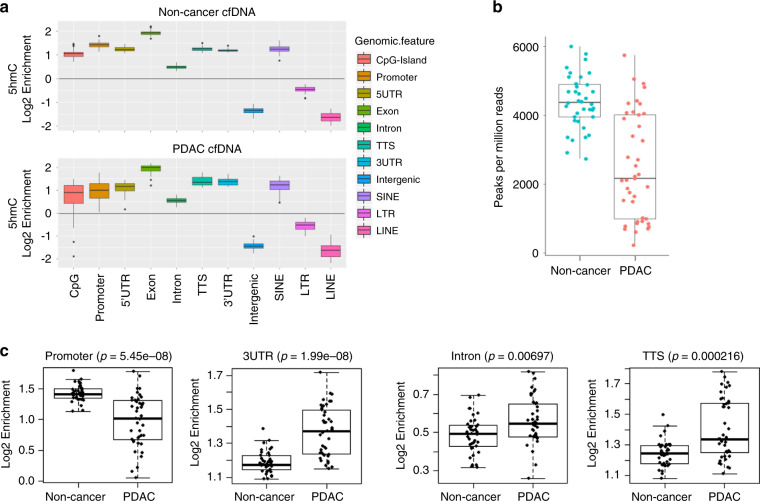
Differential enrichment of 5hmC in genomic features in PDAC cfDNA compared with non-cancer cfDNA samples. **a** Boxplots showing 5hmC peak enrichment analysis (*y*-axis = log2 enrichment) reveal that gene-based features and SINEs are enriched in 5hmC peaks in both PDAC (*n* = 41) and non-cancer (*n* = 38) cfDNA cohorts. Intergenic and LINEs are depleted of 5hmC peaks. **b** Number of 5hmC peaks detected per million reads in non-cancer (blue, *n* = 38) and PDAC (orange, *n* = 41) cohorts. Each dot depicts an individual patient sample. **c** Box plots depicting statistically significant changes of 5hmC peaks over promoters, 3′UTR, Intron and TTS regions in PDAC cfDNA (*n* = 41) compared to non-cancer cfDNA (*n* = 38). Each dot represents an individual cfDNA sample. *p*-values are from two-sided Wilcoxon test. For all boxplots, center line represents median, bounds of box represent 25th and 75th percentiles and whiskers are Tukey whiskers.

To gain an understanding of the genomic regions associated with hydroxymethylation in cfDNA, we first determined 5hmC enriched loci, as measured by increased read density and subsequent detection as peaks by MACS2^26^. The vast majority of 5hmC loci occur in non-coding regions of the genome, over introns and intergenic loci some of which overlap with SINE repetitive elements, with no preferential distribution in the PDAC or non-cancer cohort (Supplementary Fig. 1A). However, 5hmC are not particularly enriched over these regions relative to the genome background (Fig. 1a). Instead, 5hmC enrichment is observed over genic features, most significantly in promoters, 5′UTRs, 3′UTRs, exons, transcription termination sites (TTS) and SINE repetitive elements that are located in gene-rich regions, as measured by increased relative fold change compared to the genome background (Fig. 1a). These results indicate that 5hmC in cfDNA is preferentially enriched in genic regions, consistent with previously published reports^27^.

Comparison of 5hmC peaks in PDAC to the non-cancer cohort revealed significant differences. First, peaks detected per million reads in PDAC cfDNA cohort was significantly less than in non-cancer cohort (Fig. 1b). Decreased number of peaks suggests global decrease in 5hmC in PDAC, consistent with previous reports investigating tissue samples. Second, 5hmC peak enrichment was increased in PDAC over 3’UTR, TTS and intron regions whereas it was decreased over promoters (Fig. 1c). These global changes, observed in a statistically significant manner in each cohort, were also detected in various cancer stages, including early stage cancers (Supplementary Fig. 1B).

**Fig. 2:**
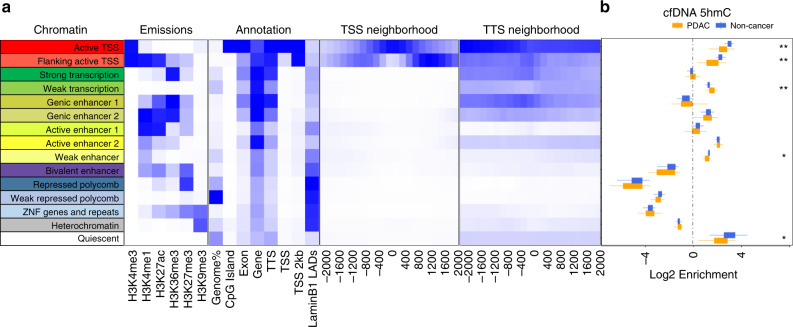
Differential 5hmC enrichment over chromatin states identified in PDAC primary tissue in PDAC cfDNA compared with non-cancer cfDNA samples. **a** Chromatin states observed in two primary PDAC tumor tissues as determined by 6 histone markers; H3K4me1, H3K4me3, H3K9me3, H3K27ac, H3K27me3, and H3K36me3. **b** Box plots depicting 5hmC occupancy in PDAC (*n* = 41) and non-cancer (NC, *n* = 38) cfDNA cohorts over the chromatin states determined in PDAC primary tissue samples. Center line represents median, bounds of box represent 25th and 75th percentiles and whiskers are Tukey whiskers. Statistical significance in PDAC vs non-cancer cfDNA enrichment, as determined by two-sided Wilcoxon test, is denoted by ** (*p* value < 0.00001) and * (*p* value < 0.0001). *p*-values are 1.85E−06 (active TSS), 1.28E−06 (flanking active TSS), 8.59E−06 (weak transcription), 4.28E−05 (weak enhancer) and 2.51E−05 (quiescent).

Next, we investigated 5hmC occupancy, and its associated changes in PDAC, with respect to chromatin state. For this purpose, we first generated histone maps of primary tumor tissues obtained from two different PDAC patients with chromatin immunoprecipitation followed by sequencing. Targeting post-translational modifications such as methylation and acetylation on histone H3 that define various functional regions, we segmented the chromatin into 15 chromatin states, that identify actively transcribed and silent regions, as well as regulatory regions, using chromHMM^28^ (Fig. 2a). In parallel, we profiled the 5-hydroxymethylome of primary PDAC tumor tissues from 17 PDAC patients and found that they overlapped most with the active TSS as well as active enhancer regions (Supplementary Fig. 2A), indicating that 5hmC marks regulatory regions with active transcription. Comparison of 5hmC occupancy in PDAC cfDNA and non-cancer cfDNA cohorts revealed statistically significant differences in these chromatin regions, with decreased 5-hydroxymethylation in PDAC cohort over active TSS and flanking TSS regions, while increased 5-hydroxymethylation was observed over weakly transcribed regions (Fig. 2b). 5hmC decrease over H3K4me3-marked active TSS sites was observed across all cancer stages (Fig. 1c and Supplementary Fig. 2B and C). These results suggest that differential 5hmC enrichment observed over promoters (Supplementary Fig. 1B) are driven by transcriptional activation. Overall, differential cfDNA hydroxymethylation over different chromatin contexts identified in tumor tissue, suggests that elements of epigenetic dysregulation in cancer cells can be picked up in the cfDNA 5hmC signal.

### Identification of disease specific genes from plasma samples

**Fig. 3:**
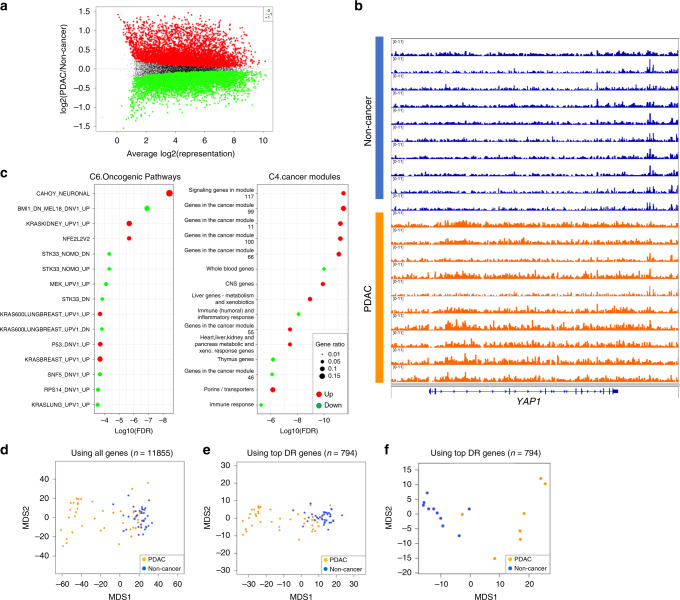
Differential 5hmC occupancy analysis in PDAC cfDNA as compared to cfDNA from non-cancer patients. **a** MA-Plot showing all genes with differential 5hmC representation. Red and green, respectively denote increased or decreased 5hmC density in PDAC compared to non-cancer with adjusted *p*-value <0.01, derived by Benjamini–Hochberg method. **b** IGV genome browser snapshot of *YAP1* locus showing the increased 5hmC signal intensity in PDAC samples compared to non-cancer controls. **c** GSEA of 794 genes with the most statistically significant differential 5hmC representation (adjusted *p*-value < 0.01, by Benjamini–Hochberg method) and filtered for fold change in 5hmC representation (|(5hmC-PDAC/5hmC-non-cancer)| ≥ 1.5) and minimum average expression (log2(average representation) ≥ 3.5) in PDAC cfDNA as compared to non-cancer samples. Log10 FDR values are derived from Kolmogorov–Smirnov test. Pathways with represented in genes with increased and decreased 5hmC are denoted with red and green, respectively. **d** MDS of pancreatic cancer (orange) and non-cancer (blue) cfDNA samples using 11,855 genes with statistically significant (adjusted *p*-value < 0.01, Benjamini–Hochberg method) increase or decrease in 5hmC. Note reasonable partitioning of PDAC from non-cancer samples. **e**, **f** MDS of pancreatic cancer (orange) and non-cancer (blue) cfDNA samples from this study (**e**) and Song et al. (**f**) using 794 genes with statistically significant differential 5hmC representation (adjusted *p*-value < 0.01, Benjamini–Hochberg method) and filtered for fold change in 5hmC representation (|(5hmC-PDAC/5hmC-non-cancer)| ≥ 1.5) and minimum average expression (log2(average representation) ≥ 3.5).

Differential analysis of 5hmC densities in genes using an adjusted *p*-value (Benjamini–Hochberg method) threshold of 0.05, revealed 5700 hyper- and 6155 hypo-hydroxymethylated genes in PDAC compared to non-cancer samples (Fig. 3a). Further filtering of this gene set using a more stringent criteria (absolute fold change ≥1.5 and average log2 CPM ≥3.5) resulted in 577 upregulated and 217 downregulated genes. Among the genes with increased 5hmC density in PDAC were those related to pancreas development (*GATA4*^29^, *GATA6*^29^, *PROX1*^30^, *ONECUT1*^31^, and *MEIS2*^32^) and/or implicated in cancer (*YAP1*^33^, *TEAD1*^33^, *PROX1*^34^, *ONECUT2*, *ONECUT1,* and *IGF1*) (Fig. 3b). Inspection of the MSigDB for cancer relevant gene sets, C6.Oncogenic signatures and C4.Cancer modules, enriched among the 577 genes with increased 5hmC densities revealed a preponderance of gene sets that are upregulated in both *KRAS* and *TP53* mutant cancers (Fig. 3c). These genes were also enriched in targets of transcription factors known to be involved in PDAC oncogenesis or metastasis, like NFAT and FOXA (HNF3) (Table 2). In contrast, the most significantly downregulated 217 genes in PDAC cfDNA cohort was enriched for gene sets that are downregulated in *KRAS* mutant cells as well as immune response and whole blood genes (Fig. 3c). These results suggest that 5hmC profiling can capture PDAC tumor relevant biological signals in plasma.

**Table 2:** Top 10 transcription factor targets enriched among 577 genes with increased 5hmC density in PDAC cfDNA versus non-cancer cfDNA. p-values were derived from Kolmogorov–Smirnov (K–S) test.

Gene set name	Description	# Genes in overlap (k)	k/K	*p*-value	FDR *q*-value
AACTTT_UNKNOWN	Genes having at least one occurrence of the highly conserved motif M17 AACTTT in the region spanning up to 4 kb around their transcription start sites. The motif does not match any known transcription factor binding site (v7.4 TRANSFAC).	117	0.0604	2.93E−40	1.79E−37
TGGAAA_NFAT_Q4_01	Genes having at least one occurrence of the highly conserved motif M55 TGGAAA sites. The motif matches transcription factor binding site V$NFAT_Q4_01 (v7.4 TRANSFAC).	101	0.0524	1.71E−29	5.22E−27
TAATTA_CHX10_01	Genes having at least one occurrence of the highly conserved motif M23 TAATTA sites. The motif matches transcription factor binding site V$CHX10_01 (v7.4 TRANSFAC).	60	0.0729	1.18E−24	2.39E−22
CTTTGA_LEF1_Q2	Genes having at least one occurrence of the highly conserved motif M73 CTTTGA sites. The motif matches transcription factor binding site V$LEF1_Q2 (v7.4 TRANSFAC).	68	0.0545	9.10E−21	1.39E−18
TTANTCA_UNKNOWN	Genes having at least one occurrence of the highly conserved motif M64 TTANTCA in the region spanning up to 4 kb around their transcription start sites. The motif does not match any known transcription factor binding site (v7.4 TRANSFAC).	59	0.0611	1.77E−20	2.16E−18
TATAAA_TATA_01	Genes having at least one occurrence of the highly conserved motif M51 TATAAA sites. The motif matches transcription factor binding site V$TATA_01 (v7.4 TRANSFAC).	65	0.0495	8.82E−18	8.97E−16
TGATTTRY_GFI1_01	Genes having at least one occurrence of the highly conserved motif M94 TGATTTRY sites. The motif matches transcription factor binding site V$GFI1_01 (v7.4 TRANSFAC).	31	0.1047	1.29E−17	1.12E−15
TGTTTGY_HNF3_Q6	Genes having at least one occurrence of the highly conserved motif M83 TGTTTGY sites. The motif matches transcription factor binding site V$HNF3_Q6 (v7.4 TRANSFAC).	46	0.0617	2.57E−16	1.96E−14
TGACATY_UNKNOWN	Genes having at least one occurrence of the highly conserved motif M42 TGACATY in the region spanning up to 4 kb around their transcription start sites. The motif does not match any known transcription factor binding site (v7.4 TRANSFAC).	43	0.064	6.84E−16	4.64E−14
CTTTAAR_UNKNOWN	Genes having at least one occurrence of the highly conserved motif M29 CTTTAAR in the region spanning up to 4 kb around their transcription start sites. The motif does not match any known transcription factor binding site (v7.4 TRANSFAC).	52	0.0524	2.16E−15	1.32E−13

Multidimensional scaling (MDS) analysis using either the 11,855 genes with high variation in 5hmC counts (Fig. 3d) or the 794 genes filtered at the extremes of 5hmC representation in PDAC (Fig. 3e), reveal partitioning of the PDAC samples from the non-cancer equally well. We then tested the partitioning of a previously published dataset^23^ using the differentially represented genes we identified. This dataset, despite small cohort size, had a similar cancer stage distribution as our discovery dataset (Supplementary Fig. 3B). Hierarchical clustering using these 794 genes revealed partitioning of the 5hmC data from PDAC and non-cancer cfDNA from Song et al.^23^ as well as the discovery cohort (Supplementary Fig. 3A, C). Consistently, PDAC samples in Song et al. could be separated from non-cancer samples using these 794 genes as shown by the MDS plot (Fig. 3f). In summary, we have been able to identify a differentially represented gene set whose biological functions are congruent with both pancreatic development and cancer. Furthermore, 5-hydroxymethylation densities of these genes alone enable the distinction of PDAC from non-cancer.

### Predictive models for detection of pancreatic cancer in cfDNA

We performed regularized logistic regression analysis in order to determine whether gene-based features are present in the PDAC and non-cancer cohorts that can enable the classification of patient samples. We employed top 65% genes with the most variable 5hmC density for model selection. Elastic net^35^ was utilized as the regularization method. Other modeling approaches, such as random forest, support vector machines and neural networks, were explored in a preliminary analysis and were found to have inferior cross-validated performance on the training data (data not shown).

**Fig. 4:**
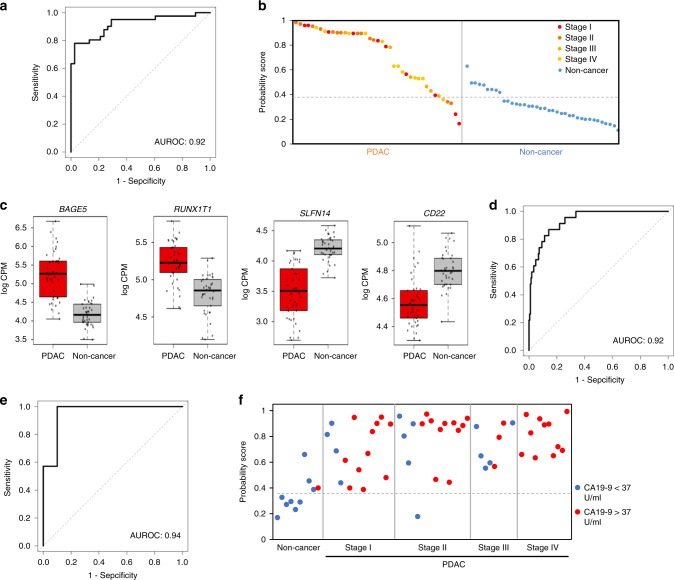
Identification of a 5hmC signature that differentiates PDAC cfDNA from non-cancer samples. **a** Predictive modeling using regularized regression model (elastic net) on the discovery dataset with 41 PDAC and 38 non-cancer cfDNA. **b** Probability scores derived for each sample in the discovery dataset using the elastic net. Probability scores towards 1 are predicted cancer samples whereas probability scores close to 0 are non-cancer samples. Dotted line denotes Q3 of the probability score of non-cancer samples. **c** 5hmC coverage (expressed in logCPM) over *BAGE5*, *RUNX1T1*, *SLFN14* and *CD22* in PDAC (*n* = 41) and non-cancer (*n* = 38) cfDNA cohorts as example of top selected model features. For all boxplots, center line represents median, bounds of box represent 25th and 75th percentiles and whiskers extend to minimum or maximum values. Each dot represents an individual cfDNA sample. **d**, **e** ROC curves for independent validation cohorts of 23 PDAC cfDNA and 205 non-cancer cfDNA processed internally (**d**) and 7 PDAC cfDNA and 10 non-cancer cfDNA from Song et al. (**e**). **f** Predicted probability scores for non-cancer (*n* = 10), and Stage I (*n* = 15), Stage II (*n* = 16), Stage III (*n* = 8) and Stage IV (*n* = 11). Same samples were also analyzed for CA19-9 levels. Samples within the clinically defined normal range (0–37 U/ml) are denoted in blue, and samples that are above are denoted in red.

Elastic net^35^ regularization method requires specifying hyper-parameters that control the level of regularization used in the fit. These hyper-parameters were selected based on out-of-fold performance on 30 repetitions of 5-fold cross-validated analysis of the training data. Out-of-fold assessments are based on the samples in the left-out fold at each step of the cross-validated analysis. The training set yielded an out-of-fold performance metric, area under the ROC curve (AUC), of 0.919 (Fig. 4a). The distribution of probability scores for each sample indicated few false negatives in the PDAC cohort and false positives in non-cancer cohort, assuming a threshold cutoff of 3rd quartile of the non-cancer (Fig. 4b). Inspection of features that were selected by the predictive model revealed cancer relevant genes, such as the cancer antigen family gene *BAGE5*^36^ and transcriptional co-repressor *RUNX1T1*^37^. On the other hand, model features that were downregulated in PDAC cfDNA compared to non-cancer cohort were enriched for immune/blood cell relevant genes such as *SLFN14* which is important for platelet formation^38^ and *CD22* which is expressed in B cells^39^ (Fig. 4c).

Next, the trained model was tested on two independent validation sets of patient samples. The first validation dataset was generated by 5hmC profiling of 23 PDAC and 205 non-cancer cfDNA samples, as described in methods section. This independent validation dataset yielded a classification performance AUC of 0.921 (Fig. 4d). A second validation set included pancreatic cancer and non-cancer samples from Song et al.^23^ which was profiled for 5hmC using a pull down method similar to the one used in this study (pancreas subtype specified as adenocarcinoma, 7 pancreas cancer, 10 non-cancer). This validation set exhibited a performance AUC of 0.943 (Fig. 4e).

### Correlation of prediction performance with CA19-9

We next investigated the performance of 5hmC-based prediction probabilities with relationship to plasma levels of CA19-9 (Cancer Antigen 19-9), which is a clinically relevant biomarker in pancreatic cancer. Despite being one of the most clinically utilized PDAC biomarkers, it is well established that CA19-9 levels in blood have challenges associated with specificity and sensitivity. Consistent with these previous observations, we found that CA19-9 level was abnormally high in one non-cancer sample (200 U/ml) and was within normal range for some Stage I, Stage II and even Stage III PDAC samples (Fig. 4f). Interestingly, high probability scores calculated by our predictive model allowed detection of these early stage, namely Stage I and Stage II PDAC samples, that had low CA19-9 levels (8 out of 30 samples) (Fig. 4f). On the other hand, probability scores of some PDAC samples were low despite high CA19-9 levels. Taken together, these results suggest that 5hmC signals can significantly improve detection on existing methods for both sensitivity and specificity, particularly for early stage PDAC.

### Prediction performance with tissue-derived features

**Fig. 5:**
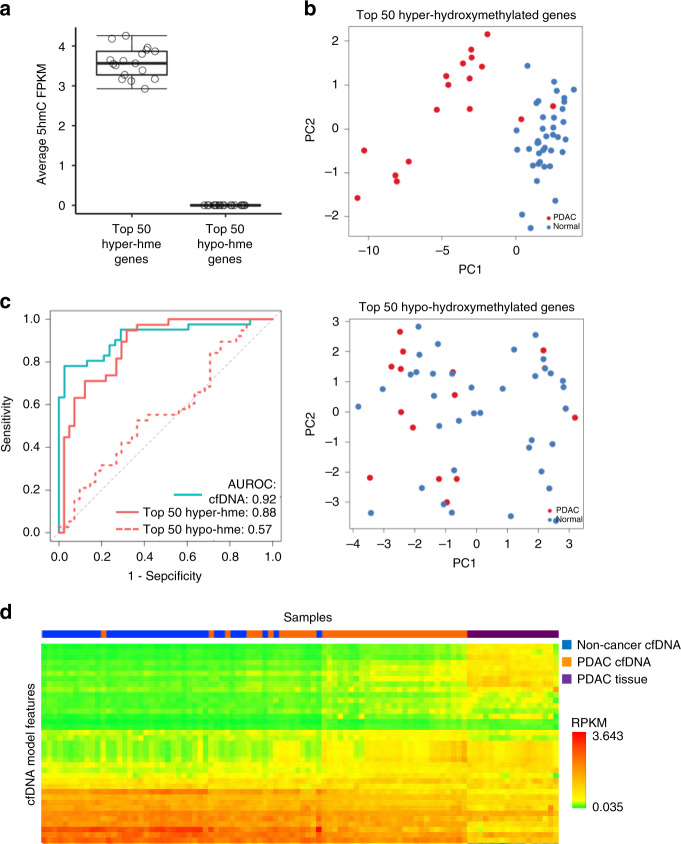
Tissue-derived 5hmC features are able to classify PDAC cfDNA. **a** Average 5hmC FPKM values over top 50 hyper-hydroxymethylated (hyper-hme) genes with the highest 5hmC occupancy compared with the top 50 hypo-hydroxymethylated (hypo-hme) genes with the lowest 5hmC occupancy in PDAC tumor tissues (*n* = 17). Boxplot center lines represent median, bounds of box represent 25th and 75th percentiles and whiskers are Tukey whiskers. Each dot represents an individual PDAC tumor tissue. **b** PCA plots showing non-cancer (blue) and PDAC (red) cfDNA cohorts using top 50 hyper-hydroxymethylated (upper panel) and top 50 hypo-hydroxymethylated (bottom panel) genes as features. **c** ROC curves for predictive model using features discovered in cfDNA (teal), top 50 genes with highest 5hmC representation in tissue (solid coral), or 50 genes with lowest 5hmC representation in tissue (dotted coral). **d** Heatmap showing FPKM values of all the gene features (*n* = 37) used in cfDNA prediction model (represented in each row) in non-cancer cfDNA, PDAC cfDNA, and PDAC tissue 5hmC profiles (represented in each column).

Next, we wanted to explore whether we can detect tumor-originated 5hmC features in cfDNA. For this purpose, we first profiled 5hmC in 17 PDAC tumor tissues. We then ranked all the genes based on FPKM values in these tissue profiles and took two gene sets; one representing the highest level of 5hmC occupancy (top 50 hyper-hydroxymethylated genes) and another set that represents the lowest levels of 5hmC occupancy (top 50 hypo-hydroxymethylated genes) in PDAC tissue (Fig. 5a). PDAC tissue-derived top 50 hyper-hydroxymethylated genes can separate non-cancer cfDNA from PDAC cfDNA samples well (Fig. 5b, top panel). In contrast, non-cancer and PDAC cfDNA samples did not cluster separately when top 50 hypo-hydroxymethylated gene set was used as features (Fig. 5b, bottom panel). Consistent with these findings, a prediction model built using hyper-hydroxymethylated gene set performed well with an AUC of 0.88 in classifying PDAC and non-cancer cfDNA samples correctly (Fig. 5c, coral line), unlike the model that used hypo-hydroxymethylated gene set as features, which had an AUC of 0.57 (Fig. 5c, dotted coral line). While the model trained with cfDNA 5hmC profiles performed best with an AUC of 0.919 (Fig. 5c, teal line), inspection of normalized 5hmC signal (RPKM) from 37 features selected in cfDNA model demonstrates that PDAC cfDNA signal is overall admixed between non-cancer cfDNA and PDAC tissue (Fig. 5d). Taken together, our results indicate that PDAC tumor tissue-derived features are useful in classification of PDAC in cfDNA, suggesting that tumor-derived epigenomic signals are retained in the cfDNA compartment.

## Discussion

Pancreatic cancer is the deadliest form of cancer with 10 percent five-year survival rate^40^. A major factor behind such abysmal survival rate is the difficulty in diagnosing patients early when tumors can be surgically removed. This study was focused on the discovery of hydroxymethylation-based biomarkers in cfDNA that may facilitate the development of molecular diagnostic tests to detect pancreatic cancer at not only late but also early stages. 5hmC-based methods were previously reported to have potential for cancer detection from plasma samples, particularly in the context of lung cancer, hepatocellular carcinoma^23^, colon and gastric cancer^25^. Furthermore, differential 5hmC profiles, albeit from limited number of pancreatic cancer patients, suggested such an approach could be possible for pancreatic cancer^23^. Early stage detection from plasma have proved to be difficult for approaches that depend solely on tumor originating DNA, such as mutational analysis, due to minute levels of circulating tumor DNA present at early stage disease. Methods such as DNA methylation or hydroxymethylation profiling, on the other hand, has the potential to leverage all signals in the plasma, including the ones that originate from immune cells, a major contributor of cfDNA^41^, which can, in turn, improve detection for early stage cancers. Indeed, DNA methylation-based methods have recently shown promise in cancer detection^42,43^. Our data show that pancreatic cancer detection at early stages is possible with 5hmC-based methods.

Our data highlight the ability to detect differentially hydroxymethylated genes whose underlying biology shows association with both pancreas and cancer development as well as established trends in chromatin mark maps and other functional regions of the genome. Furthermore, regularized regression was used to build a predictive model from a comprehensive gene set that is highly variable, yielding an AUC of 0.919 along with two independent external dataset validations with AUC of 0.921 and 0.943.

The 5hmC signal was readily found to overlap in gene-centric functional regions (enrichment in promoter, exons, UTR and TTS), as well as transposable elements like SINEs (enriched) and LINEs (depleted) (Fig. 1a). Globally, PDAC cfDNA cohort had decreased number of peaks compared to non-cancer cfDNA cohort (Fig. 1b), consistent with previous reports of decreased 5hmC in several types of cancer, including pancreatic cancer^44^. Indeed, decreased 5hmC was recently linked to malignant transformation in *KRAS* mutant pancreatic cells upon deactivation of p53, which are commonly observed in PDAC patients^45^. Hydroxymethylcytosine changes in functional regions have also been reported in cfDNA from colorectal^25^, esophageal^24,46^ and lung cancer^24^. Consistent with these reports, we observed decreased number of peaks in PDAC cfDNA relative to non-cancer cfDNA. Furthermore, we report PDAC specific gains or losses in hydroxymethylation in functional regions in our data. PDAC specific 5hmC increase in 3’UTR, TTS and exons and 5hmC decrease in promoters detectable in cfDNA (Fig. 1c). These changes were also observed in various pancreatic cancer stages (Supplementary Fig. 1). In embryonic stem cells, 5-hydroxymethylation decreases in the promoter region have been shown to associate with elevated gene transcription^27^. An increase in disease relevant transcription is implicitly supported in our PDAC data by the 5hmC increase in gene-centric features mentioned earlier, as well as an apparent decrease of 5hmC in promoter regions (Fig. 1c). Taken together, disease specific remodeling of active demethylation in PDAC patients is captured via changes in 5hmC representation.

Dynamic changes in chromatin have been shown to control cell development and transition of cells with oncogenic potential^47^. Intersection of our 5hmC data with various chromatin states determined by ChIPseq in PDAC primary tumor tissues revealed 5hmC localization in active chromatin regions, most significantly active TSS and active enhancer regions (Fig. 2a). Consistent with 5hmC changes over promoters, 5hmC decrease in PDAC cfDNA in active TSS regions also suggests disease specific increases in gene transcription via chromatin modifications, given the permissive transcriptional state associated with H3K4me3^48^. Furthermore, we observed 5hmC decrease in weak enhancer regions identified by H3K27ac and H3K4me1 (Fig. 2a). While 5hmC patterning around known functional elements of the genome suggests a broader interplay between hydroxymethylation and the epigenetic control of transcriptional processes, these results also indicate that 5hmC in cfDNA can potentially be utilized for non-invasive monitoring of epigenomic dysregulation in PDAC. Additional work will reveal the extent to which models predictive of PDAC can be built from a combination of gene-specific features, genomic loci with different chromatin states and transposable elements detected in cfDNA.

In this study, we employed aggregate quantification of hydroxymethylation at gene level in PDAC, and yet, were able to find genes and other functional regions with changes in 5hmC signals that highlighted pathways implicated in pancreatic cancer (Fig. 3c). The majority of PDAC cancers harbor activating mutations in *KRAS* (~90%) and inactivating mutations in *TP53* (~70%)^49^. Gene set enrichment analysis for the genes with increased 5hmC representation in gene body revealed several gene sets that are upregulated when KRAS is up or when p53 is down (Fig. 3c left panel). Furthermore, among genes with increased 5hmC were targets of transcription factors NFAT and FOXA (HNF3) (Table 2), previously reported to be involved in promoting pancreatic cancer initiation^50^ and metastasis^51^, respectively, via enhancer reprogramming. Investigation of genes with decreased 5hmC in PDAC cfDNA as compared to non-cancer cfDNA indicated enrichment of genes downregulated when KRAS is up. Genes related to whole blood and immune response were enriched among the genes with decreased 5hmC in PDAC cfDNA (Fig. 3c right panels). This would be consistent with an increase in (tumor) tissue derived DNA in cfDNA in PDAC patients, diluting immune and blood cell derived DNA that make up the majority of cfDNA in non-cancer individuals^41^. These results, taken together, suggest that PDAC tissue derived signals can be detected in cfDNA from cancer patients using 5hmC.

Inspection of individual genes that were either significantly increased or decreased in 5hmC density revealed genes implicated in normal pancreas development, for instance the transcription factors *GATA4*, *GATA6*, *PROX1*, *ONECUT1*/*2*, in addition to genes whose increased expression is implicated in cancer, such as *YAP1*, *TEAD*, *PROX1*, *ONECUT2*, *ONECUT1* and *IGF1*. The relative 5hmC increase in transcription factor genes like *GATA4*, *GATA6*, *PROX1*, *ONECUT1*/*2*, *MEIS2*, which were previously reported to be involved in early pancreatic development^29–31^, suggest a reversion to a stem-like state in PDAC samples.

Genes with the most significant increase in 5hmC in PDAC cfDNA are enriched in annotated relevant biology which can be used to build regularized regression models with a high performance (training AUC of 0.919 with external dataset validation AUCs of 0.921 and 0.943). This gives us good confidence that our models are measuring underlying biological signals relevant to PDAC. One such signal is the cancer antigen *BAGE* that is selected among the 37 features in our model.

Despite the large number of differentially hydroxymethylated genes identified in PDAC cfDNA compared to non-cancer cfDNA, the regularized regression model with only 37 genes was sufficient to perform well for classification of cfDNA. However, 13,180 differentially hydroxymethylated genes detected in PDAC cfDNA compared to non-cancer cfDNA suggest that other biological signals may also reside in our dataset. Future work is needed to understand the impact of other biological factors on differential hydroxymethylation in PDAC cfDNA.

Smoking status is a known risk factor for PDAC up to 20 years post smoking cessation and DNA methylation changes have been associated with tobacco-based toxins^52^. In our prospective case–control designed study, ever smokers constituted 51.2% and 55.3% of PDAC and non-cancer cohorts respectively, indicating that ever smokers are well represented in each cohort. Consequently, we do not believe that smoking association in our PDAC cohort could account for the significantly hydroxymethylated genes found. Indeed, statistical adjustment for genes affected by smoking results in comparable performance for predictive models (data not shown). However, a more extensive future study focused on sub-partitioning PDAC and non-cancer patient into never and ever smokers with pack-year characteristics will enable us to address the impact of smoking on the hydroxymethylome in PDAC patients. Furthermore, high specificity is crucial to achieve in the clinical setting for detection of cancers with low incidence rate such as pancreatic cancer. Pancreatic cancer risk parameters combined into a clinically relevant, intent-to-test population-based study, will allow testing of our findings beyond our current case–control cohort study, which numbers less than 100 participants. Further consideration of disease-related clinical parameters will enable us to explore hydroxymethylcytosine features with the aim of yielding refined signals capable of earlier diagnosis of PDAC.

## Methods

### Clinical cohorts and study design

A case–control study was performed using plasma obtained from subjects without (termed non-cancer) and with pancreatic cancer. Patients were enrolled from participating sites in the United States at which informed consent was obtained and biospecimens collected as approved by the Institutional Review Boards (IRBs) responsible at each site (Sterling IRB or WIRB, Supplementary Table 1). The study protocol submission, IRB approval and specimen handling across all sites were managed by MT Group (Van Nuys, CA), who provided clinical research specimen handling services.

### Cancer cohort

Plasma samples for the cancer cohort were obtained from subjects who had undergone management for pancreatic cancer in the United States, and also provided consent for use of blood specimens for archival storage and retrospective analyses.

Criteria for subject eligibility for inclusion in the analysis included male and female subject age of minimum 40 years old with a tolerance of 5% of patients younger than 40 years old, with additional requirements for the cancer cohort including: (1) no cancer treatment, e.g., surgical, chemotherapy, immunotherapy, targeted therapy, or radiation therapy, prior to study enrollment and blood specimen acquisition; and (2) a confirmed pathologic diagnosis of adenocarcinoma inclusive of all subtypes.

### Non-cancer cohort

Subject exclusion criteria for the non-cancer cohort also included any of the following: prior cancer diagnosis within prior six months; surgery or invasive procedure requiring general anesthesia within prior month; non-cancer systemic therapy associated with molecularly targeted immune modulation; concurrent or prior pregnancy within previous 12 months; history of organ tissue transplantation; history of blood product transfusion within one month; and major trauma within six months. Clinical data required for all subjects included age, gender, smoking history, and both tissue pathology and grade, and were managed in accordance with the guidance established by the Health Insurance Portability and Accountability Act (HIPAA) of 1996 to ensure subject privacy.

### Plasma collection

Plasma was isolated from whole blood specimens obtained by routine venous phlebotomy at the time of subject enrollment. For both cancer and non-cancer control subjects, whole blood was collected in Cell-Free DNA BCT® tubes according to the manufacturer’s protocol (Streck, La Vista, NE) (https://www.streck.com/collection/cell-free-dna-bct/). Tubes were maintained at 15 °C to 25 °C with plasma separation performed within 24 h of phlebotomy by centrifugation of whole blood at 1600 x g for 10 min at RT, followed by transfer of the plasma layer to a new tube for centrifugation at 16,000 x g for 10 min. Plasma was aliquoted for subsequent cfDNA isolation or storage at −80 °C.

### cfDNA isolation

cfDNA was isolated using the QIAamp Circulating Nucleic Acid Kit (QIAGEN, Germantown, MD) following the manufacturer’s protocol excepting the omission of carrier RNA during cfDNA extraction. Four milliliter plasma volumes were lysed for 30 min prior to collection of nucleic acids. Eluates were collected in a volume of 60 µl buffer. All cfDNA eluates were quantified by Bioanalyzer dsDNA High Sensitivity assay (Agilent Technologies, Santa Clara, CA) and Qubit dsDNA High Sensitivity Assay (Thermo Fisher Scientific, Waltham, MA) was employed to ensure the absence of contaminating high molecular weight DNA emanating from white blood cell lysis.

### Tissue and genomic DNA processing

All tissue samples were stored in H media for the interval between surgical resection and laboratory processing. Each sample was weighed and aliquoted into sections of approximately 35 mg. Each resulting subsection was briefly incubated on dry ice, then homogenized in 500 µl RLT Buffer Plus using a Tissue Lyser LT (QIAGEN Germantown, MD) at 50 Hz for two minutes. Resulting homogenates were stored at −80 C until DNA extraction. Genomic DNA was extracted using DNeasy Blood & Tissue Kit (QIAGEN Germantown, MD) according to the manufacturer instructions. Genomic DNA eluates were quantified using the Qubit dsDNA High Sensitivity assay (Thermo Fisher Scientific, Waltham, MA) and stored at −20 °C until further processing. Prior to sequencing library construction, genomic DNA was fragmented to a modal 150 base pair size using an ME220 focused ultrasonicator (Covaris, Woburn, MA), Modal fragmented DNA sizes were verified using the TapeStation 2200 dsDNA high sensitivity assay (Agilent Technologies, Santa Clara, CA) and quantified as described above prior to commencing library construction.

### 5-hydroxymethyl Cytosine (5hmC) enrichment assay

^35^cfDNA was normalized to 10 ng total input for each assay and ligated to sequencing adapters. 5hmC bases were biotinylated via a two-step chemistry and subsequently enriched by binding to Dynabeads M270 Streptavidin (Thermo Fisher Scientific, Waltham, MA). All libraries were quantified by Bioanalyzer dsDNA High Sensitivity assay (Agilent Technologies, Santa Clara, CA) and Qubit dsDNA High Sensitivity Assay (Thermo Fisher Scientific, Waltham, MA) and normalized in preparation for sequencing.

### DNA sequencing and alignment

DNA sequencing was performed according to manufacturer’s recommendations with 75 base-pair, paired-end sequencing using a NextSeq550 instrument with version 2 reagent chemistry (Illumina, San Diego, CA). Data were collected using NextSeq System Suite 2.2.04. Twenty-four libraries were sequenced per flowcell. Raw data processing and demultiplexing was performed using the Illumina BaseSpace Sequence Hub to generate sample specific FASTQ output. Sequencing reads were aligned to the hg19 reference genome using BWA-MEM with default parameters^53^. Sequencing data quality was assessed using picard^54^.

### Peak detection

BWA-MEM read alignments were employed to identify regions or peaks of dense read accumulation that mark the location of a hydroxymethylated cytosine residue. Prior to identifying peaks, BAM files containing the locations of aligned reads were filtered for poorly mapped (MAPQ < 30) and not properly paired reads using samtools and HTSlib^55^. 5hmC peak calling was carried out using MACS2 (https://github.com/taoliu/MACS) with a *p*-value cut off = 1.00e−5. Identified 5hmC peaks residing in “blacklist regions” as defined elsewhere (https://sites.google.com/site/anshulkundaje/projects/blacklists) and residing on chromosomes X, Y, and mitochondrial genome were also removed using bedtools^56^. Computation of genomic feature enrichment overlap 5hmC peaks were performed using HOMER software (http://homer.ucsd.edu/homer/) with default parameters.

### Chromatin immunoprecipitation

Chromatin immunoprecipitations of H3K4me1, H3K4me3, H3K27ac, H3K36me3, H3K9me3, and H3K27me3 in primary PDAC tumor tissues were performed at Active Motif (Carlsbad, CA). Briefly, tumor tissues were homogenized, then sonicated and subjected to chromatin immunoprecipitation with antibodies specific to chromatin modifications mentioned above (anti-H3K4me1 Active Motif 39297, 4 μl per IP; anti-H3K4me3 Active Motif 39159, 3 μl per IP; anti-H3K9me3 Abcam ab8898, 5 μl per IP; anti-H3K27ac Active Motif 39133, 4 μg per IP; anti-H3K27me3 Active Motif 39155, 4 μg per IP; anti-H3K36me3 Active Motif 61101, 4 μg per IP). Immunoprecipitated DNA was isolated, then subjected to the library preparation and was subsequently sequenced. Reads were mapped using BWA-MEM, then filtered for quality reads as described above. Peaks for each histone modification was determined using MACS2 with default parameters for H3K4me1, H3K4me3, and H3K27ac; while –broad option was used for H3K9me3, H3K27me3, and H3K36me3. ChromHMM was run with all 6 histone ChIPseq mentioned above^28^. For comparisons between PDAC and non-cancer, two-sided Wilcoxon test was used, and for across stages comparison, two-sided Kruskal–Wallis test was employed. Genomic regions were visualized using IGV^57^.

### Differential representation analysis

For the purpose of reliably identifying gene bodies with differential representation between the PDAC and the non-cancer groups, we closely followed the RNA-Seq workflow outlined in Law et al.^58^, including much of the preliminary QC steps, using R^59^. In brief, the analysis includes data pre-processing by adopting the following workflow: (i) transforming the data from raw counts to log2(counts per million), (ii) removing genes that are weakly represented, (iii) normalizing the gene representation distributions, and (iv) performing unsupervised clustering of samples. To accomplish differential representation analysis, we applied the following steps: (i) creating a design matrix to contrast PDAC versus non-cancer cohorts, (ii) removing heteroscedasticity from the data, (iii) fitting regression models for the comparison of interest, PDAC vs non-cancer, (iv) examining the number of differentially represented genes. In most of these analysis steps the default settings were used when appropriate. To remove weakly represented genes, we excluded genes that did not have greater than 3 counts per million reads in at least 20 samples. This filter excludes roughly 12% of the genes. For the identification of the significantly differentially represented regions, we used the method of Benjamini and Hochberg^60^ to obtain *p*-values adjusted for multiple comparisons. In this report, we use adjusted *p*-value and false discovery rate (FDR) interchangeably.

### Predictive modeling

For the purpose of assessing the feasibility of building classifiers that can discriminate between PDAC and non-cancer samples based on the 5hmC representation of gene bodies, we used elastic net, which is commonly used in the classification context, where the number of examples are few and the number of features are large. See Friedman et al.^35^ for a description of the general Elastic net procedure. Software implementation of these methods can be found at https://cran.r-project.org/web/packages/glmnet/index.html. Weakly represented genes were excluded from analysis as described in the section on “Differential representation analysis”.

All training and fitting were done on 80% of the samples selected at random in a balanced way to keep the ratio of the number of PDAC to non-cancer samples similar in both the training and testing subsets. Before any fitting, genes were filtered to include the top 65% of the most variable genes for model fitting task. The filter was designed using training samples only and was done in a way to ensure that genes of all levels of 5hmC representation were included.

Hyperparameters of the regularization model were selected based on out-of-fold performance on 30 repetitions of 5-fold cross-validated analysis of the training data. Out-of-fold assessments are based on the samples in the left-out fold at each step of the cross-validated analysis. The out-of-fold performance of the models fitted with hyperparameter values set at the optimal values might yield a slightly optimistic assessment of performance. The performance of these models applied to the test set should provide less biased estimate of performance, although generalizability to external datasets is not always guaranteed.

The hyperparameter values that lead to the best out-of-fold performance were then used to fit the final models which were fitted to the entire set of samples, including both training and testing subsets. The performance of these final models can thus only be evaluated based on their performance on external datasets. These do provide a sense of the generalizability of the performance observed in the local training and testing datasets.

To evaluate the effect of feature selection on prediction performance, we repeated the training and evaluation task based on a filtered set of genes that included genes found to be significantly differentially represented, having a 1.5-fold differential 5hmC representation, and a level of representation exceeding the median level (log2 CPM ≥ 3.5). This filter was designed based on training data statistics only.

### CA19-9 detection

CA 19-9 was detected from plasma by electrochemiluminescent immunoassay (Roche) at Arup Laboratories.

### Reporting summary

Further information on research design is available in the Nature Research Reporting Summary linked to this article.

## Supplementary information

Supplementary Information

Reporting Summary

## Data Availability

Processed gene counts and BED files data from this study can be accessed from NCBI Gene Expression Omnibus under accession number GSE152137. The Fastq files can be made available upon written request for submission to the study institutional review boards at the participating sites (Sterling IRB or WIRB) for approval, please contact the corresponding author with requests. The remaining data is available in the Article, Supplementary Information.
