# Supplementary material for: Detection of early stage pancreatic cancer using 5-hydroxymethylcytosine signatures in circulating cell free DNA
Source: Nat Commun. 2020 Oct 19;11:5270. doi: 10.1038/s41467-020-18965-w (PMC7572413; doi:10.1038/s41467-020-18965-w)
Supplement: Supplementary file 1 — Supplementary Information [file 41467_2020_18965_MOESM1_ESM.pdf]

Detection of early stage pancreatic cancer using 5-hydroxymethylcytosine signatures in circulating cell free DNA

Guler et al

Supplementary Figures S1-S3

Supplementary Table S1

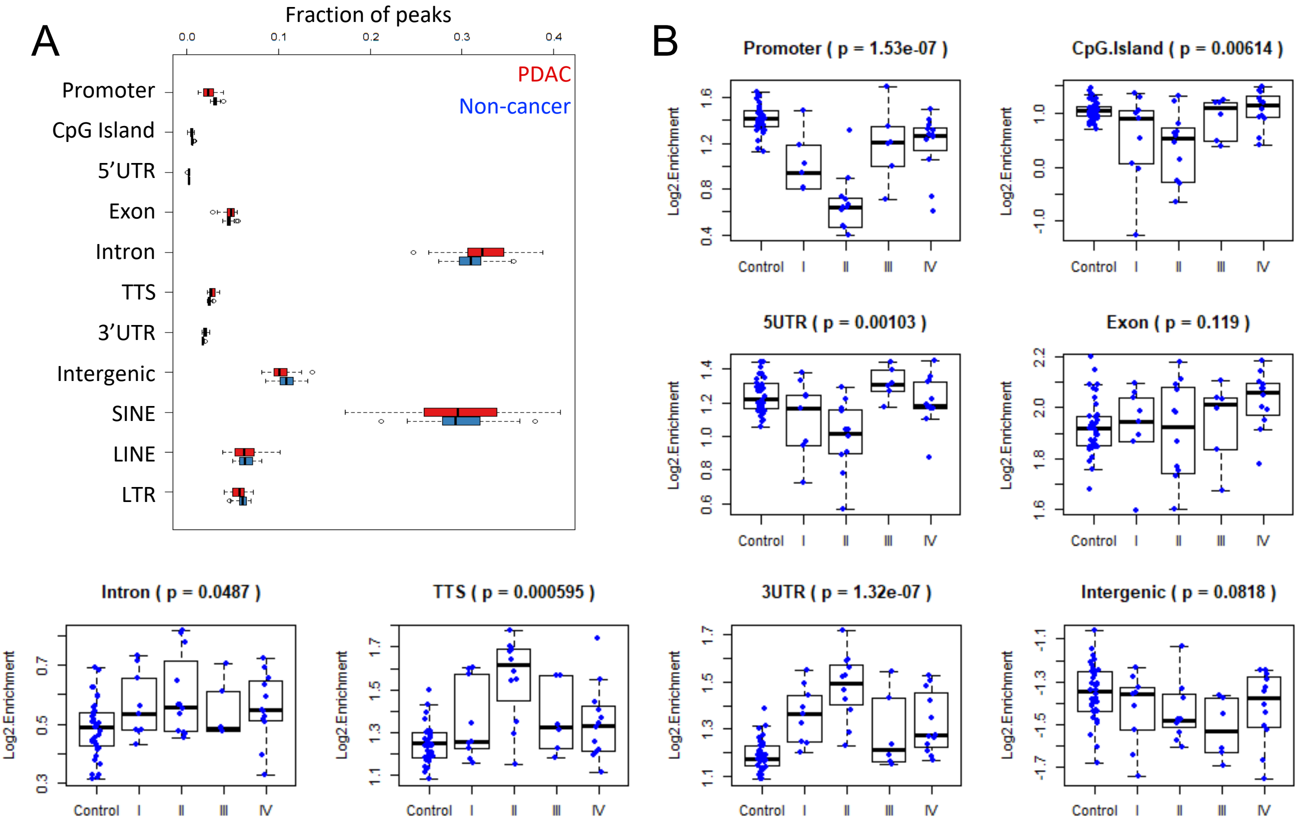

Supplementary Figure 1. Differential enrichment of 5hmC in genomic features in PDAC cfDNA compared with non-cancer cfDNA samples.

a. 5hmC peak distribution over genomic features in PDAC (n=41) and non-cancer (n=38) cfDNA.

b. 5hmC log2 enrichment over functional genomic regions in PDAC (n=41) and non-cancer (n=38) cfDNA cohorts calculated over genomic background. Box plots depicting statistically significant changes of 5hmC peaks in functional genomic regions across pancreatic cancer stages. p-values show statistical significance by two-sided Kruskal-Wallis test.

For all boxplots, center line represents median, bounds of box represent 25th and 75th percentiles and whiskers are Tukey whiskers.

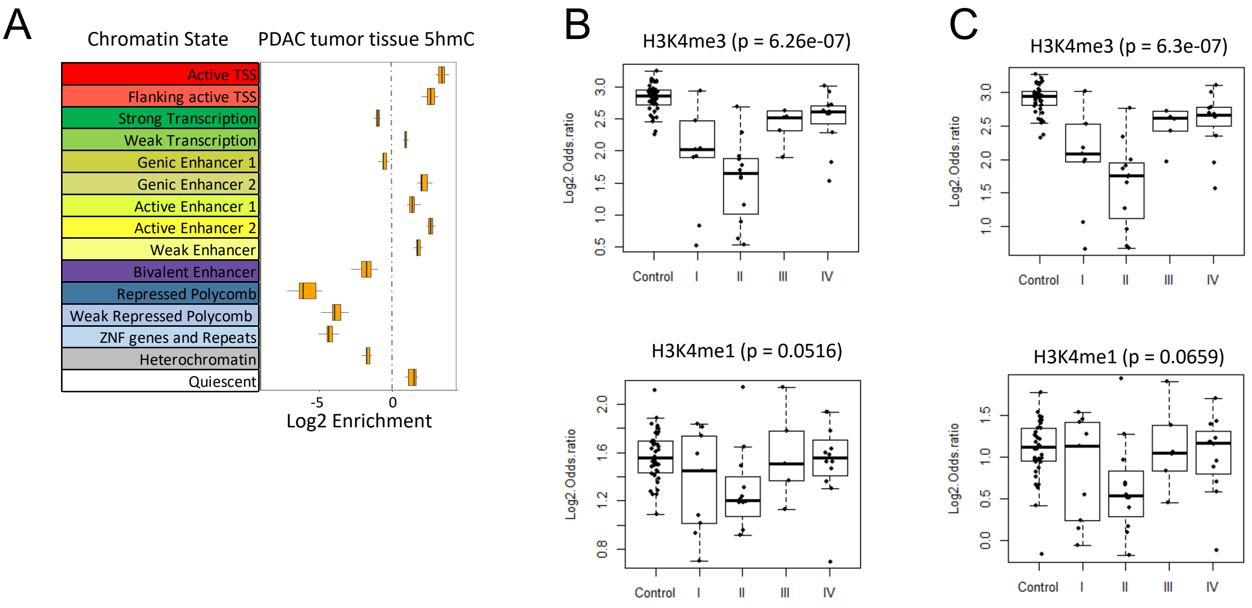

Supplementary Figure 2. Differential 5hmC enrichment in PDAC cfDNA compared to non-cancer cfDNA over chromatin states identified in PDAC primary tissues**.**

a. Log2 enrichment of 5hmC peaks identified in PDAC primary tumor tissues (n=17) over chromatin states of PDAC primary tumors identified by chromHMM.

b-c. Boxplot depicting cfDNA 5hmC log2 enrichment across pancreatic cancer stages as compared to non-cancer controls over H3K4me3 and H3K4me1 occupied genomic regions as determined in PDAC primary tumor tissue 1 (b) and 2 (c) from two individual patients. Each dot in boxplot represents an individual cfDNA sample. p-values show statistical significance by two-sided Kruskal-Wallis test.

For all boxplots, center line represents median, bounds of box represent 25th and 75th percentiles and whiskers are Tukey whiskers.

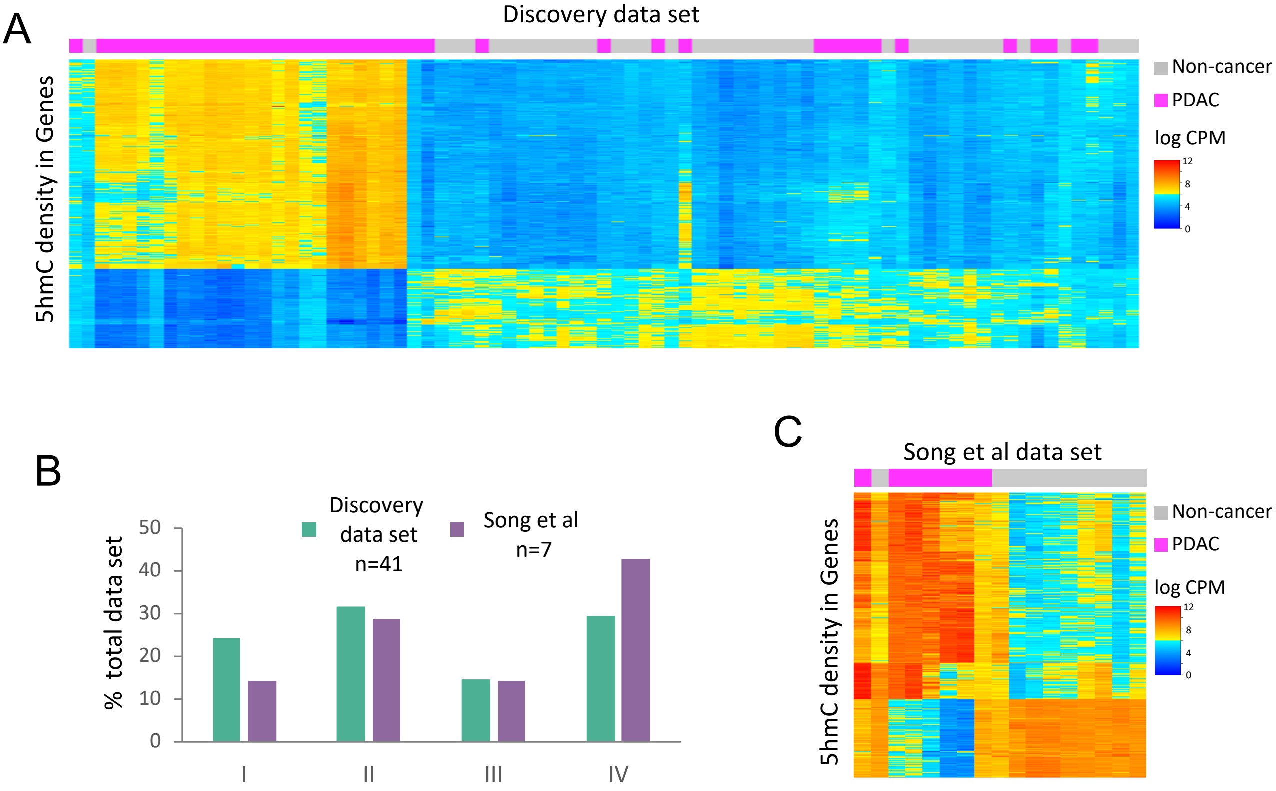

Supplementary Figure 3. Partitioning of PDAC and non-cancer cfDNA samples using a small set of differentially hydroxymethylated genes.

a. Hierarchical clustering of pancreatic cancer (magenta) and healthy (gray) cfDNA samples from discovery cohort employing 5hmC counts over 794 differentially hydroxymethylated genes identified in this study.

b. Distribution of patients over pancreatic cancer stages I-IV from this study (teal) and Song et al. (purple) expressed as percentage of total cohort in each study.

c. Hierarchical clustering of pancreatic cancer (magenta) and healthy (gray) cfDNA samples from Song et al study employing 5hmC counts over 794 differentially hydroxymethylated genes identified in this study.

Table S1. List of IRBs for each participating sample collection site.

Site Number	Site IRB	# of Pancreas Cancer Samples	# of Control Samples
54	Sterling	10	
98	Sterling	3	
38	Sterling	30	
82	Sterling	2	14
94	Sterling	1	
93	Sterling	1	
92	Sterling	1	
112	Sterling	1	
50	WIRB	1	
118	WIRB	2	
83	WIRB	6	
99	Sterling		81
105	Sterling		40
106	Sterling		37
122	Sterling		71
103	Sterling	6	
