# Supplementary material for: Detection of early stage pancreatic cancer using 5-hydroxymethylcytosine signatures in circulating cell free DNA
Source: Nat Commun. 2020 Oct 19;11:5270. doi: 10.1038/s41467-020-18965-w (PMC7572413; doi:10.1038/s41467-020-18965-w)
Supplement: Supplementary file 2 — Reporting Summary [file 41467_2020_18965_MOESM2_ESM.pdf]

nature research

Corresponding author(s): Samuel Levy

Last updated by author(s): Aug 18, 2020

Reporting Summary

Nature Research wishes to improve the reproducibility of the work that we publish. This form provides structure for consistency and transparency in reporting. For further information on Nature Research policies, see our Editorial Policies and the Editorial Policy Checklist.

Statistics

For all statistical analyses, confirm that the following items are present in the figure legend, table legend, main text, or Methods section.

n/a	Confirmed	
☐	☒	The exact sample size (*n*) for each experimental group/condition, given as a discrete number and unit of measurement
☐	☒	A statement on whether measurements were taken from distinct samples or whether the same sample was measured repeatedly
☐	☒	The statistical test(s) used AND whether they are one- or two-sided<br>*Only common tests should be described solely by name; describe more complex techniques in the Methods section.*
☐	☒	A description of all covariates tested
☐	☒	A description of any assumptions or corrections, such as tests of normality and adjustment for multiple comparisons
☐	☒	A full description of the statistical parameters including central tendency (e.g. means) or other basic estimates (e.g. regression coefficient) AND variation (e.g. standard deviation) or associated estimates of uncertainty (e.g. confidence intervals)
☐	☒	For null hypothesis testing, the test statistic (e.g. *F*, *t*, *r*) with confidence intervals, effect sizes, degrees of freedom and *P* value noted<br>*Give P values as exact values whenever suitable.*
☒	☐	For Bayesian analysis, information on the choice of priors and Markov chain Monte Carlo settings
☒	☐	For hierarchical and complex designs, identification of the appropriate level for tests and full reporting of outcomes
☒	☐	Estimates of effect sizes (e.g. Cohen's *d*, Pearson's *r*), indicating how they were calculated

Our web collection on statistics for biologists contains articles on many of the points above.

Software and code

Policy information about availability of computer code

Data collection	NextSeq System Suite 2.2.04
Data analysis	Software used for data analysis in this study are: BWA 07.17-r1188, Samtools 1.9, htslib 1.9, bedtools v2.29.2, Picard 2.22.3, R 3.5.0, MACS2 2.1.2, HOMER v4.10, IGV 2.4.15

For manuscripts utilizing custom algorithms or software that are central to the research but not yet described in published literature, software must be made available to editors and reviewers. We strongly encourage code deposition in a community repository (e.g. GitHub). See the Nature Research guidelines for submitting code & software for further information.

Data

Policy information about availability of data

All manuscripts must include a data availability statement. This statement should provide the following information, where applicable:

- Accession codes, unique identifiers, or web links for publicly available datasets
- A list of figures that have associated raw data
- A description of any restrictions on data availability

Processed data from this study can be accessed from NCBI Gene Expression Omnibus under accession number GSE152137. Remaining data is included in the manuscript figures, tables and methods section. Fastq files can be made available upon written request for submission to the study institutional review board for approval.

April 2020

Field-specific reporting

Please select the one below that is the best fit for your research. If you are not sure, read the appropriate sections before making your selection.

[x] Life sciences [ ] Behavioural & social sciences [ ] Ecological, evolutionary & environmental sciences

For a reference copy of the document with all sections, see nature.com/documents/nr-reporting-summary-flat.pdf

Life sciences study design

All studies must disclose on these points even when the disclosure is negative.

Sample size	From previous studies (Song et al 2017, Li et al 2017) sample sizes in the range of 10-50 patients per cohort produced a differential 5hmC pattern. Therefore, we based our collection of samples within the high portion of this range.
Data exclusions	There were no data exclusions.
Replication	We have completed two replications in our manuscript and verified the results of our study. These datasets were pancreas and non-cancer data sets from Song et al 2017 and an additional independent dataset that we processed in-house.
Randomization	This was a case-control study design therefore allocation of samples into groups were non-random. However, we examined smoking status as a covariate and found that it did not account for the detected signal in distinguishing each cohort.
Blinding	Because our study was a discovery study, blinding was not relevant.

Reporting for specific materials, systems and methods

We require information from authors about some types of materials, experimental systems and methods used in many studies. Here, indicate whether each material, system or method listed is relevant to your study. If you are not sure if a list item applies to your research, read the appropriate section before selecting a response.

Materials & experimental systems

n/a	Involved in the study	
[ ]	[x]	Antibodies
[x]	[ ]	Eukaryotic cell lines
[x]	[ ]	Palaeontology and archaeology
[x]	[ ]	Animals and other organisms
[ ]	[x]	Human research participants
[x]	[ ]	Clinical data
[x]	[ ]	Dual use research of concern

Methods

n/a	Involved in the study	
[ ]	[x]	ChIP-seq
[x]	[ ]	Flow cytometry
[x]	[ ]	MRI-based neuroimaging

Antibodies

Antibodies used	Following antibodies were used for ChIPseq:<br>• anti-H3K4me3: Active Motif 39159<br>• anti-H3K4me1: Active Motif 39297<br>• anti-H3K27me3: Active Motif 39155<br>• anti-H3K36me3: Active Motif 61101<br>• anti-H3K27ac: Active Motif 39133<br>• anti-H3K9me3: Abcam ab8898
Validation	• anti-H3K4me3: Active Motif 39159: dot blot by Active Motif, ChIP-qPCR at known positive and negative control sites, dot blot and ChIP by the Antibody Validation Database (Egelhofer et al, 2010).<br>• anti-H3K4me1: Active Motif 39297: dot blot by Active Motif, ChIP-qPCR at known positive and negative control sites, dot blot and ChIP by the Antibody Validation Database (Egelhofer et al, 2010).<br>• anti-H3K27me3: Active Motif 39155: dot blot by Active Motif, ChIP-qPCR at known positive and negative control sites, dot blot and ChIP by the Antibody Validation Database (Egelhofer et al, 2010).<br>• anti-H3K36me3: Active Motif 61101: Validated for ChIPseq by Active Motif; dot blot and ChIP-qPCR at known positive and negative control sites.<br>• anti-H3K27ac: Active Motif 39133: dot blot by Active Motif, ChIP-qPCR at known positive and negative control sites, dot blot and ChIP by the Antibody Validation Database (Egelhofer et al, 2010).<br>• anti-H3K9me3: Abcam ab8898: Peptide competition assay by Abcam, ChIP-qPCR at known positive and negative control sites, dot blot and ChIP by the Antibody Validation Database (Egelhofer et al, 2010).<br>Reference:

Egelhofer et al, An assessment of histone-modification antibody quality, Nature Structural & Molecular Biology, 2010, doi:10.1038/nsmb.1972

Human research participants

Policy information about studies involving human research participants

Population characteristics	Please see table 1 in our manuscript for a description of population characteristics of the human research participants in our study.
Recruitment	Recruitment of cancer patients and non-cancer patients were prospectively collected by a third party under their controlled IRB protocol. No selection bias was observed.
Ethics oversight	Cancer and non-cancer samples were collected under the MT group IRB protocol.

Note that full information on the approval of the study protocol must also be provided in the manuscript.

ChIP-seq

Data deposition

- [x] Confirm that both raw and final processed data have been deposited in a public database such as GEO.
- [ ] Confirm that you have deposited or provided access to graph files (e.g. BED files) for the called peaks.

Data access links<br>*May remain private before publication.*	Deposited data can be accessed on Gene Expression Omnibus (GEO) with accession number GSE152137.
Files in database submission	Tissue2_ChrStates.bed<br>Tissue1_ChrStates.bed
Genome browser session<br>(e.g. UCSC)	No longer applicable.

Methodology

Replicates	We performed biological replicates using two primary PDAC tissue samples taken from two individual patients.
Sequencing depth	Tissue1_K4me1: Total number of reads= 96641391, Uniquely mapped reads= 69612147, 75 bp paired end<br>Tissue1_K4me3: Total number of reads= 34593168, Uniquely mapped reads= 31297615, 75 bp paired end<br>Tissue1_K9me3: Total number of reads= 97672807, Uniquely mapped reads= 84813889, 75 bp paired end<br>Tissue1_K27ac: Total number of reads= 91353340, Uniquely mapped reads= 53308145, 75 bp paired end<br>Tissue1_K27me3: Total number of reads= 110638515, Uniquely mapped reads= 62254633, 75 bp paired end<br>Tissue1_K36me3: Total number of reads= 104636247, Uniquely mapped reads= 81864888, 75 bp paired end<br>Tissue1_input: Total number of reads= 76242032, Uniquely mapped reads= 68939494, 75 bp paired end<br>Tissue2_K4me1: Total number of reads=91196620, Uniquely mapped reads=69116459, 75 bp paired end<br>Tissue2_K4me3: Total number of reads= 43830559, Uniquely mapped reads= 39564005, 75 bp paired end<br>Tissue2_K9me3: Total number of reads= 76686263, Uniquely mapped reads= 63236643, 75 bp paired end<br>Tissue2_K27ac: Total number of reads= 100724588, Uniquely mapped reads= 49465946, 75 bp paired end<br>Tissue2_K27me3: Total number of reads= 88163498, Uniquely mapped reads= 31103200, 75 bp paired end<br>Tissue2_K36me3: Total number of reads= 106614154, Uniquely mapped reads= 77911172, 75 bp paired end<br>Tissue2_input: Total number of reads= 90127298, Uniquely mapped reads= 82444847, 75 bp paired end
Antibodies	• anti-H3K4me3: Active Motif 39159<br>• anti-H3K4me1: Active Motif 39297<br>• anti-H3K27me3: Active Motif 39155<br>• anti-H3K36me3: Active Motif 61101<br>• anti-H3K27ac: Active Motif 39133<br>• anti-H3K9me3: Abcam ab8898
Peak calling parameters	Command line used for read mapping: bwa mem -M -t genome.fa R1.fastq R2.fastq<br>Command line used for peak calling: macs2 callpeak -t 5hmC.bam -c wgs.bam -f BAMPE -g hs --pvalue 0.00001 ```
Data quality	Aligned reads were filtered to remove poorly mapped (MAPQ < 30) and not properly paired reads. 5hmC peak calling was carried out using MACS2 (https://github.com/taoliu/MACS) with a p-value < 1e-05. ENCODE blacklist regions were also removed. Below is the number of peaks per experiment that contained 5 fold enrichment at less than 0.05% FDR.<br>Tissue1_K27ac: 110691<br>Tissue1_K27me3: 47967<br>Tissue1_K36me3: 135009<br>Tissue1_K4me1: 107777<br>Tissue1_K4me3: 20242

	Tissue1_K9me3: 27635<br>Tissue2_K27ac: 105451<br>Tissue2_K27me3: 25891<br>Tissue2_K36me3: 165395<br>Tissue2_K4me1: 145318<br>Tissue2_K4me3: 21453<br>Tissue2_K9me3: 12194
Software	BWA 07.17-r1188, Samtools 1.9,
